# Smokers’ and Young Adult Non-Smokers’ Perceptions and Perceived Impact of Snus and E-Cigarette Modified Risk Messages

**DOI:** 10.3390/ijerph17186807

**Published:** 2020-09-18

**Authors:** Olivia A. Wackowski, Mariam Rashid, Kathryn L. Greene, M. Jane Lewis, Richard J. O’Connor

**Affiliations:** 1 Rutgers Center for Tobacco Studies, Rutgers Biomedical and Health Sciences, Rutgers University, New Brunswick, NJ 08901, USA; mrashid3@sph.rutgers.edu (M.R.); lewismj@sph.rutgers.edu (M.J.L.); 2 Department of Communication, Rutgers University, New Brunswick, NJ 08901, USA; klgreene@rutgers.edu; 3 Department of Health Behavior, Rowell Park Comprehensive Cancer Center, Buffalo, NY 14263, USA

**Keywords:** tobacco harm reduction, communication, modified risk tobacco products, snus, smokeless tobacco, e-cigarettes, product perceptions, tobacco regulatory science

## Abstract

Although no tobacco products are safe, tobacco companies in the United States may request regulatory authorization to make certain “modified risk tobacco product” (MRTP) claims in their marketing. However, few qualitative studies have explored consumer perceptions and understanding of comparative risk messages and wordings. We examined consumer perceptions of statements indicating reduced risks and exposure to chemicals from snus and e-cigarettes relative to smoking. We conducted 12 focus groups with adult smokers (ages 21–66) and young adult (YA) non-smokers (ages 18–25) (*n* = 57) in the USA in 2019. Participants shared reactions to modified risk and exposure messages and message variations. Participants largely understood claims, including language about “switching completely.” However, participants expressed desire for more message specificity, evidence, risk reduction reasons, and statistics/quantitative information. Claim believability and acceptance was also limited by existing negative product beliefs and experiences, negative media reports, and skepticism about message source. YAs did not express product interest based on message exposure. Some YAs suggested the included “smoker” language made the messages less interesting/relevant. Given existing proposals for and use of MRTP messages, additional research on their wording, framing, delivery, and effects may help inform regulatory or organizational decisions about such messages, optimize potential benefits, and mitigate unintended consequences.

## 1. Introduction

Scientific experts and reviews suggest that certain types of non-combusted tobacco and nicotine products, such as smokeless tobacco (e.g., snus and moist snuff) and various electronic nicotine delivery systems and vaping devices (referred to here as “e-cigarettes”) pose lower levels of risk to users than cigarette smoking (though they are not safe or without risks), and might serve as harm reduction alternatives for smokers who switch to them completely [1,2,3,4,5]. Although quitting all tobacco and nicotine products is the safest course of action, tobacco harm reduction approaches may be appropriate to help smokers who are unable or unwilling to quit altogether or quit with traditional cessation methods [6]. Research suggests that some smokers may be receptive to products communicated as posing lower risks than cigarettes [7,8].

In the United States, tobacco companies are prohibited from making any types of comparative risk or “modified risk tobacco product” (MRTP) claims in their marketing, unless they submit applications to make such claims to the Food and Drug Administration (FDA) under a new regulatory pathway, and receive authorization to make such claims. These could include claims about modified or reduced risk of disease or harm to health, and/or reduced risk of exposure (e.g., to some constituent) [9]. However, the regulatory review imposes high standards for MRTP status—MRTP claims must be accurate and backed by science, and applications should include evidence that consumers understand and are not misled by the claims. Furthermore, evidence should demonstrate that MRTP marketing could reduce harm in smokers who switch to the products, without simultaneously introducing population-level harm that outweighs this benefit, such as product uptake by non-tobacco users including young adults and youth [9].

To date applications from three smokeless tobacco brands (General Snus, Camel Snus, Copenhagen) have been submitted to the FDA with one of these (General Snus) authorized thus far (October 2019) [10]. The modified risk claims proposed by these brands have varied in a number of ways, including, for example, the number of reduced health risks named (ranging from just one to six listed health effects) and the presence or absence of a qualifier suggesting degree of harm reduction (e.g., “can *greatly* reduce risk”). And while Camel and Copenhagen’s claims make use of wording signaling the claim is directed at smokers (e.g., “if you smoke, consider this…”) and state that reduced risks come from “switching completely,” General Snus claim does not make this explicit. This is important given that harm reduction appears to be conditional on completely switching (and not dual product use) [11]. However, the limited research on this wording so far is somewhat mixed on whether consumers understand what it means [12,13,14]. Camel’s proposed ads also include modified exposure claims (e.g., stating that “scientific studies have shown that Camel Snus contains less of the harmful chemicals than cigarette smoke”) as well as several statements attempting to connect the role of combustion with modified risk and exposure (e.g., “no smoke = less risk”).

Although no MRTP proposals have yet been submitted for e-cigarettes, one has been submitted for the electronic heated tobacco product IQOS. Although the FDA denied two IQOS proposed claims suggesting risk/harm reduction in July 2020, the FDA authorized one claim about reduced exposure to chemicals (“Scientific studies have shown that switching completely from cigarettes to the IQOS system significantly reduces your body’s exposure to harmful or potentially harmful chemicals.”) [15]. Similar proposals could conceivably be made in the future for e-cigarettes and would also require research and scrutiny. Indeed, Health Canada has already conducted research on a set of modified risk and exposure statements for potential authorized use by industry [14]. Furthermore, research suggests the public may already be exposed to e-cigarette modified risk claims—one study found that 29% of smokers indicated seeing an e-cigarette claiming to be “less harmful” [16]. These may be unauthorized industry claims or messages from other sources. Public health organizations such as the US Centers for Disease Control and Public Health England have also included some messages about reduced harm or exposure from e-cigarettes in their communications [5,17]. As such, it is important to know how people perceive and respond to such messages.

Qualitative research is important for exploring consumer perceptions and understanding of comparative risk messages and potential variations for developing new ones [6]. However, limited such investigation exists outside of the industry’s own research [12,14,18]. This is important given recommendations for independent MRTP studies outside of the industry [6]. Furthermore, findings from the limited previous studies are somewhat mixed. For example, one qualitative study about e-cigarette comparative claims with smokers concluded that messages emphasizing the harms of smoking could be effective in motivating smokers to quit and/or switch to e-cigarettes [12], while another found that comparative statements emphasizing smoking harms were more likely to be perceived as minimizing the negative health effects of vaping, particularly among young non-smokers [14]. Research on authorized claims is also relevant because MRTP status is not permanent and can change or be revoked if post-market surveillance finds negative outcomes (e.g., claim misunderstanding, youth uptake, etc.) [9].

Given ongoing proposals for MRTP messages and some existing use of such messages, this study aims to contribute to the new literature on MRTP claim understanding and perceptions with a qualitative study exploring both smokers’ and young adult non-smokers’ reactions to claims about snus and e-cigarettes. This data may help to inform relevant message development, refinement and/or message evaluation. We focus on snus given it is a more “traditional” product proposed for harm reduction, for which evidence exists about its reduced health risks compared to cigarettes [2,3], and on e-cigarettes as a class of more recently developed products that appear to have consumer appeal and harm reduction potential [4,5], but for which research about its health risks in general and compared to cigarettes is still evolving. We conducted focus groups with current adult smokers, given they are the primary intended audience of MRTP messages for tobacco harm reduction, as well as with young adult non-smokers, as young adults have been identified as a priority group for studies of MRTP marketing and potential unintended consequences of such marketing (such as product appeal and uptake) [6,9].

## 2. Materials and Methods

### 2.1. Participants and Recruitment

We conducted twelve small in-person focus groups—six focused on snus claims and six on e-cigarette/vaping claims. Within each cluster of six groups, four included adult current smokers (i.e., have ever smoked 100 cigarettes and now smoke every day or some days) and two were with young adult (YA) non-smokers (i.e., 8 groups with current smokers and four with YA non-smokers). Across groups we aimed to recruit participants who were not current regular users of e-cigarettes or smokeless tobacco, although they may have used these in the past or occasionally. Additional inclusion criteria included being at least 18 years old and able to read and speak English. We aimed to keep the groups small and conduct these as “mini-focus groups” (with 3–6 participants each) [19,20,21], given our intent for each participant to discuss reactions to multiple messages (*n* = 57 total participants, 32 smokers, 25 YA non-users).

Most participants identified as female (65%), 12% were Hispanic and half (50%) were white (with 23% Asian, 16% Black/African American, 11% other race). About 37% had at least a college degree, 32% had some college or technical school education, and 30% had a high school degree or GED as their highest completed education. The average age of smokers was 44 (range 21–66) and age 20 for YAs (range 18–25). Most smoker participants (84%) smoked daily and most (80%) had been smoking for over 10 years. In addition, most (81.3%) smokers and 56% of YA participants had tried e-cigarettes/vaping products before. About 34% of smokers and 20% of YAs had vaped in the past 30 days. Fewer had ever tried any smokeless tobacco (23% smokers, 8% YAs).

Participants were recruited from ad postings in the central New Jersey section of the classified advertisements website, Craigslist, flyer postings on the Rutgers University college campus and surrounding neighborhoods, and recruitment e-mails sent through Rutgers employee personnel listservs. Consent letters were sent to participants prior to group sessions and presented again at the beginning of sessions. Groups were conducted between August and October 2019.

### 2.2. Message Stimuli and Group Procedures

Focus groups were approximately 90 min long and began with questions about participants’ exposure to and experience with cigarettes as well as smokeless tobacco and/or e-cigarettes (depending on group). Next, participants viewed and provided feedback on a series of e-cigarette or snus MRTP messages (depending on group). Each group viewed and rated a primary or “base” modified risk claim (focused on reduction to health harms) and a “base” modified exposure claim (focused on reduction of chemicals in the product) (see Table 1). To facilitate discussion, participants were asked to rate each base message on a scale of 1–10 about (1) how clearly it communicated that the product is less harmful than smoking if you are a smoker, (2) whether it is clear and easy to understand, (3) how clearly it communicates that there are still harms associated with the product, and (4) how much it would encourage them to completely switch to the product (in smoker groups) or start using the product (in YA groups). Following discussion of each base claim, participants viewed and discussed several wording variations of that claim. Although there were differences in the specificity of the modified risk claim for snus and e-cigarettes (with risk reductions listed for specific diseases only for snus), the overall message structure and wording variations presented across snus and e-cigarette groups was similar/parallel (Table 1).

Messages were presented to participants one at a time on an overhead projector and in individual participant packets. Study procedures and rating measures were adapted from a study conducted for Health Canada exploring reactions to concept e-cigarette/vaping MRTP messages [14]. The messages, wording variations, and focus group guides were also informed by that previous study, as well as review of other MRTP message studies, the claims submitted in USA MRTP applications to date (see Supplementary Table S1), and feedback from interviews with 10 research experts in the tobacco control field.

After discussion, participants were debriefed that the statements were concept messages and not approved for use. The PI reviewed known risks about snus and vaping products, emphasized that potential harm reduction benefits are conditional on switching completely (not dual-use), and provided a list of smoking cessation resources. Because the e-cigarette groups were in progress during the national outbreak of e-cigarette or vaping product use-associated lung injury (EVALI) in the fall of 2019 [22], debriefing in these groups also consisted of a handout and discussion about EVALI information and recommendations from FDA and CDC at that time. Each participant was given a $50 gift card for their participation.

### 2.3. Data Analyses

Sessions were transcribed and preliminary research memos and thematic and content codes were drafted based on initial iterative transcript readings. Drafts of the results were refined upon coding of the transcripts by research team members (OW, MR) using Atlas.ti qualitative software and reviews of the coded text and previous results drafts. Illustrative quotes were selected (in some cases edited for brevity and clarity) to exemplify emergent themes and response patterns. This study was reviewed and approved by the Rutgers University Institutional Review Board (Pro2019001654).

## 3. Results

Although a few smoker participants across groups still currently both smoked and vaped, most had only tried e-cigarettes or discontinued using them after some brief trial period. Perceptions based on these experiences included not liking their taste, not deriving sufficient satisfaction from e-cigarettes, and finding them to be expensive. Some also noted that e-cigarettes were more complicated to use or too “gimmicky”, noting that others used them to “look cool” but that they were not the “real thing.” Several smokers also mentioned stopping vaping after experiencing some respiratory symptoms or other negative effects, such as throat burning:

“…But by the end of the week I noticed physical feeling in my lung area that I did not like at all….And believe it or not smoking a cigarette never made me feel that way inside. So it was a little scary.”

*(e-cigarette group, female smoker)*

Most YAs had also tried vaping before, including with friends, but some also described not liking the taste or feeling of vaping. YAs also cited concerns about addiction and other health effects as reasons for preventing them from continuing or progressing to regular cigarette smoking or vaping (including observed effects on their breathing from vaping, and hearing about other negative effects in the news, including EVALI). Some simply did not find vaping interesting or appealing. Few YAs had used smokeless tobacco before and generally thought it was “gross.”

### 3.1. Modified Risk (MR) Claim Reactions

#### 3.1.1. Clear Communication That Snus Is Less Harmful

Across smoker and YA snus groups, many agreed that the presented snus MR claim was clear and straightforward in communicating that snus is less harmful than smoking and rated it highly on this outcome. Some also had positive initial reactions to the message (e.g., “*It gave me a lot of hope….*”, snus group, female smoker). Some found some of the information to be new or surprising. This included information about reduced oral cancer risk, which seemed to be the opposite of what participants had always heard or believed. However, for some this also felt less believable:

“It can still give you things in your mouth, I know that. So, that statement is not right.”

*(snus group, male smoker)*

Some were also skeptical whether the snus message was true, even if they thought the message was clear. This included questions regarding who the message source was, and a desire for evidence and facts, including how much less harmful snus is, and what is meant by “greatly reduce”. Some also noted that the message did not explain how or why snus is less harmful (see Table 2, Section A).

#### 3.1.2. Clear Communication That Vaping Is Less Harmful

In comparison to the snus claim, smokers in the e-cigarette groups rated the viewed e-cigarette MR claim less favorably in terms of how well it communicated that e-cigarettes/vaping is less harmful than smoking. Some provided low ratings because they perceived the statement to be too general, with no specific types of harms mentioned, statistics, magnitude of reduction, or rationale for harm reduction (Table 2, Section A):

“It doesn’t have enough substance on my end to say it’s going be less harmful for me.”

*(e-cigarette group, male smoker)*

For many participants across smoker and YA e-cigarette groups, their scores and message perceptions appeared to be driven by the perceived believability of the statement. For some, message believability was based on the novelty of e-cigarettes and not enough research known yet *(“…it just hasn’t been around long enough. In my opinion, how the heck do they know?”—e-cigarette group, male smoker)*. Message believability was also related to frequent comments across groups that vaping was just as harmful as smoking cigarettes, or not really less harmful in a meaningful way. Some articulated that these perceptions were influenced by the inclusion of chemicals in e-cigarettes and by both products containing nicotine, which was perceived as being harmful (see Table 2, Section B).

Some participants also made comments suggesting beliefs that e-cigarettes may be even more harmful than cigarettes because they were causing acute symptoms and leading to death quickly. These were based on vaping risks, serious cases of addiction, and illnesses they had heard reported in the media, including the EVALI outbreak (see Table 2, Section C). However, some participants did indicate that they thought the statement was believable, explaining that cigarette smoking is very dangerous and that some have already benefitted from switching to vaping.

#### 3.1.3. Communication That Snus/Vaping Still Contains Risks

Participants across groups were mixed in their reactions as to whether the MR statement they viewed conveyed that there are still harms associated with vaping or using snus (i.e., not risk-free). Several participants across groups agreed the statements did communicate this, because the statements referred to reduced risk, which implied some harm or risk was still attached to product use:

“…at the end it says it reduces the harms. It doesn’t say it eliminates them. So you can’t say that they’re not telling you that there are still harms.”

*(e-cigarette group, female YA)*

However, participants in the e-cigarette groups also noted that that statement made vaping seem relatively harmless and did not suggest what the harms of vaping could be. Similarly, participants in the snus groups indicated that the claim presented snus in a positive way and did not emphasize or clearly communicate about its risks. In general, these comments were more pervasive in the YA groups:

“So [the claim] does dictate that it’s not going to completely get rid of all of your risk… But I don’t think it’s really hitting home that there are still negative consequences that come with this.”

*(snus group, female YA)*

#### 3.1.4. Motivation to Switch Completely/Start Using Snus or E-Cigarettes

In the snus groups, some smokers described more of an openness to trying snus based on the MR statement. However, across groups, perceived interest in completely switching (among smokers) or starting to use e-cigarettes or snus (among young adults) based on the MR message was very low overall. Lack of interest among smokers was related to the same issues described previously including beliefs that e-cigarettes/snus are still harmful (even if claim is true) and/or not meaningfully less harmful than smoking, general negative perceptions about smokeless tobacco (including that it is “gross”), and previous negative or unsatisfying experiences with e-cigarettes. Several smokers also mentioned that they enjoyed smoking, could not see themselves switching to a different product at this point in their life, and/or that they did not want to trade one vice or addiction for another (e.g., *“I’m not gonna start another habit at age 53,” e-cigarette group, male smoker*).

Several smokers commented that they thought the vaping statement could make vaping more attractive to non-smokers and young people. However, interest in initiating e-cigarette or snus use based on the message was even lower among YAs than smokers. Low interest was related to previously mentioned concerns/beliefs about product risks and perceptions that the statement was not directed at them as non-smokers. This was influenced in part by the use of smoker-directed language (e.g., “if you smoke”) in the statements:

“When I first saw that it says, ‘If you smoke,’ I was like, ‘Okay. So this does not apply to me, so I don’t care.”

*(e-cigarette group, female YA)*

### 3.2. Perceptions of Modified Risk (MR) Claim Wordings and Variations

#### 3.2.1. Switching Completely Phrasing

When asked what participants thought was meant by the phrasing “switching completely” in the MR message, participants across groups appeared to correctly understand the meaning:

“That you cannot be using snus and using cigarettes at the same time. It’s like you have to give up cigarettes and you will exclusively just use this product so that you get the benefit of it”

*(snus group, female smoker)*

When asked about replacing “switching completely” with the phrase “completely replacing” (Table 1), several participants noted that this phrasing meant or had the same impact on them, although some noted that “completely replacing” made the meaning of the statement more clear/overt. However, some smokers noted that they would be more open to the statement with the “switching completely” phrasing because it felt more “friendly” and less directive:

“Switching, it kind of gives me a choice. Opposed to the other one was more final.”

*(snus group, female smoker)*

Smoker participants were also asked for their reactions to replacing “switching completely” with “quitting smoking and switching to snus/vaping…” (Table 1). This wording was consistently perceived across smoker groups as more confusing and rejected. The direct juxtaposition of the wording and ideas of quitting smoking and taking up vaping or snus seemed incongruent and “oxymoronic”:

“I mean, you’re not really quitting if you’re going to vaping.”

*(e-cigarette group, female smoker)*

#### 3.2.2. Use of “Greatly”

Smokers were also asked their thoughts on excluding the adjective “greatly” from the MR claim they viewed (see Table 1) and provided mixed responses. Although some thought use or exclusion of “greatly” did not make a difference, several, particularly in the snus groups, noted that its inclusion was important (see Table 3, Section A). However, in both snus and e-cigarette groups, some thought the word could be misleading or give false hope and questioned its believability and evidence base. Similarly, some stated the claim was more believable and honest/realistic without the word “greatly” (Table 3, Section B). Several smokers in the snus group also preferred alternate wording that switching to snus puts you at “a much lower risk” for the stated diseases for the same reason (i.e., more modest and believable).

#### 3.2.3. Health Conditions Present in Snus MR Claim

Smokers were asked about the number of conditions presented in the snus claim, whether some should be prioritized for brevity, and whether it should also include stroke. In general participants appeared to favor having all the relevant conditions listed because it provided “more information” that way (and more reasons to switch), they were all “different” and important diseases, and that mention of different diseases may have salience for various audiences (“for each one of them, you draw a different crowd”). Participants also supported adding stroke in because this was novel information *(“It hit me when I read that last one, stroke, and I was like, ‘Wow.’”—snus group, male smoker*).

#### 3.2.4. Attribution to “Scientific Studies”

Participants were also asked their perceptions of the statement when reworded to indicate that that the stated harm reductions had been demonstrated in “scientific studies” (Table 1). While several said it would make no difference, others said that it might improve the credibility and their perception of the claim. However, some viewed the use of the term more negatively, suggesting if felt more like a sales pitch. It also raised skeptical questions about the sources of the studies, which was perceived as an important factor for believability (Table 3, Section C, D). Young adults noted that use of the phrase did not increase their product interest.

#### 3.2.5. With Emphasis on Smoking Harms

Participants were asked to view the MR statement again with a second part that emphasized the risks of smoking (“Smoking is deadly. Think seriously about switching,” Table 1). Across smoker and YA groups, some thought this addition improved the claim, made it “more serious,” attention-getting and motivating (Table 3, Section E). Some YAs also thought this version made the message feel more clearly directed at smokers. However, other smokers noted that it would make them simply think more about quitting cigarettes/nicotine completely, rather than switching to another product. Still, others thought it made no difference or rejected it, thinking it was too strong, something they already knew as smokers, and somewhat hypocritical if coming from a tobacco/nicotine company (Table 3, Section F). However, some noted that this message might work better if from a more credible source such as the CDC:

“I think it’d make a big difference….Because if they are going out of their way to say a message like this and attack smoking, then snus must be a much better alternative in which there is less risk of everything overall. So for them to come out and say this would be a big deal.”

*(snus group, male YA)*

#### 3.2.6. With Combustion Explanation

Participants were also shown the MR claim with another supplemental part, which provided a rationale for the risk reduction by referring to a lack of combustion/smoke (e.g., “Why? Because there is no tobacco burning or smoke with [Snus/vaping products]”, Table 1). Across snus and e-cigarette groups, several participants thought the addition improved the message by making it more specific or providing rationale, while others thought it was overly obvious and unnecessary. Some also thought it was somewhat misleading or not helpful because they did not consider combustion/smoke as being a main or only cause of smoking health risks:

“…You’re kind of looking at it and saying ‘Oh, so because you’re burning tobacco or blowing smoke, that’s what’s causing the cancer or the lung disease?’ Nothing else? There’s a lot more that causes it by use of the cigarette…”

*(snus group, female smoker)*

In the snus groups, some also thought the focus on smoke was not relevant to oral cancer, while in the e-cigarette groups, some thought it was not an appropriate message because e-cigarettes also release a type of “smoke”:

“When you vape with either product, there is still a burning and smoking that’s coming from it, regardless…”

*(e-cigarette group, female smoker)*

Some groups were shown a second example of an explanation statement which referred to a reduction in chemicals (i.e., why? Because there’s no smoke and fewer chemicals with snus.) (Table 1). Participants in these groups seemed to generally react more favorably to this version of the explanatory statement because it seemed more impactful (*“People are so afraid of chemicals*”, *female smoker*) and more novel/less obvious than the smoke statement.

### 3.3. Modified Exposure (ME) Claim Reactions

#### 3.3.1. Clear Communication That Snus/Vaping Is Less Harmful

As with Claim 1, many agreed across groups that the presented ME claim (focused on reduced chemicals, Table 1) was clear and straightforward in communicating that using snus or e-cigarettes is less harmful than smoking. Some thought the reference to “scientific studies” made it strong (*“anything that is backed up by science I am persuaded by,” female smoker*) and several participants described that the reference to less harmful chemicals suggested to them that the product is less harmful (Table 4, Section A). However, across snus and e-cigarette groups (including YA groups) several also noted that the claim was somewhat vague because it did not specify which chemicals were fewer nor how much lower, and if that would make a difference on health impact (Table 4, Section B). Some viewed it as a more general statement that did not necessarily communicate less harm:

“I don’t think it clearly communicates that it’s less harmful and just says it exposes you to less chemicals. That doesn’t make it any less harmful, in my opinion.”

*(e-cigarette group, female smoker)*

Some participants in the e-cigarette groups also questioned whether e-cigarettes could really even have fewer chemicals than tobacco cigarettes because tobacco is “natural” unlike e-cigarettes, which are based entirely on chemicals (Table 4, Section C).

Although several participants in the snus groups thought this ME claim was more effective than the MR claim (because it provided more rationale/explanation, or because it was shorter/more direct), more participants expressed that they thought this claim was weaker than the MR claim because it lacked the same connection to actual health impact:

“I think it’s more obvious in the first one. This isn’t as powerful. Yes, it’s fewer harmful chemicals, but what does that mean as far as my health? So, I think I like the other one better.”

*(snus group, female smoker)*

However, perceptions of the ME claim were more mixed in the e-cigarette groups. While some thought the e-cigarette ME claim was less direct in communicating reduced harm than the MR claim, others indicated that they thought it was a more “truthful” and/or believable statement than the MR claim.

#### 3.3.2. Communication That Snus/Vaping Still Contains Risks and Motivation to Switch/Start Using

Several participants across groups agreed that the ME statement communicated that there is still harm associated with the product because they understood that “fewer chemicals” did not mean “no chemicals,” which suggested the products could still be harmful. However, as described earlier, others commented that the statement was vague in that it did not specify what chemicals were in the product, or what their harms could be. Perceived impact of the ME statement on their motivation to switch to/start using the products continued to be low, with reasons similar to those expressed for the MR claim (e.g., dislike of product, belief that product was still harmful/addictive, etc.).

#### 3.3.3. Perceptions of ME Claim Variation

Participants were shown an alternative version of the ME claim that stated snus/e-cigarettes had fewer chemicals than the “7000 in cigarette smoke” (Table 1). Some thought this version was more serious, would give people pause, and potentially make some more open to switching (Table 4, Section D). However, some said this version would be more powerful in making them want to quit smoking in general, rather than switch to vaping/using snus. And others across groups noted that it did not improve the message because they did not believe there were 7000 chemicals in cigarette smoke and this was just a “scare tactic”, and/or because it still did not say how many chemicals were present in snus/e-cigarettes. Some, particularly young adults, noted this version could also backfire because it might suggest to readers that there are many chemicals (even thousands) in snus/e-cigarettes (which they might not have considered before and made the product less appealing) (Table 4, Section E).

## 4. Discussion

The promotion of tobacco harm reduction strategies is not universally accepted and may confer various benefits and risks that should be considered. Whether or not products such as snus and e-cigarettes actually promote harm reduction continues to be researched and debated in the tobacco control field. Notably, in some countries, the sale of these products is not even allowed. Nevertheless, in some countries, including the United States, modified risk claims about non-cigarette products have been proposed or are already in use, and it is important to research responses to these types of messages.

Overall, this study found that current smokers and young adult non-smokers largely understood the meaning of presented MRTP claims for snus and e-cigarettes, including language about “switching completely”, which was preferred over other alternative phrasings. However, while some smokers expressed an openness to trying snus or e-cigarettes, understanding of claims did not automatically translate into message believability or acceptance, nor to substantial product interest.

Findings were consistent with limited previous qualitative research in terms of skepticism about whether the products really pose a reduced risk, based on existing beliefs and concerns about not wanting to switch to another tobacco/nicotine product because it would still contain nicotine and/or chemicals, and still be harmful [12,14,18]. These concerns are understandable, as these products do still pose risks and can indeed maintain nicotine addiction. Furthermore, as noted by participants, the long-term risks of e-cigarette use are still unknown. These concerns are also consistent with arguments that have been made by some researchers and public health organizations in statements opposing or warning about proposed MRTP products and messages [23,24,25].

Our findings also demonstrated new concerns about e-cigarettes in particular, based on negative media reports including those about the EVALI outbreak in the fall of 2019. These well-publicized events clearly influenced participants’ believability of the e-cigarette claims as well as their product interest. It is not known to what extent smokers may have learned since then that EVALI cases were largely attributed to e-cigarettes/vapes containing tetrahydrocannabinol (THC) [22], rather than traditional nicotine e-liquids or products such as JUUL, nor to what extent concerns about EVALI related illnesses and deaths may still linger. However, in the context of growing beliefs about e-cigarette harms [26], future e-cigarette communications may need to address the EVALI outbreak and correct any misperceptions surrounding it.

Study results may also suggest that if MRTP messages are to promote harm reduction for smokers, consumers may need better education and understanding about nicotine and the relative role of combustion in the exposure to harmful chemicals and health consequences associated with smoking [27,28,29]. Indeed, participants expressed interest in more information about how and why snus and e-cigarettes could pose reduced risks, and several responded favorably to claim versions that attempted to include a “rationale” section. Understanding the role of combustion and chemicals in tobacco-related diseases may also help consumers understand why e-cigarettes appear likely to be less harmful, even if long-term studies confirming this are not yet available.

Findings were also consistent with previous research in terms of audience desire for specificity, evidence, and facts/statistics [12,14,18]. As such, the more specific snus modified risk claim (which named reduced health effects) was generally perceived as more impactful than the general e-cigarette MR claim about reduced harm to “health”. Participants also expressed a desire for quantitative information about “how much” these risks could be reduced, and what is meant by “greatly reduce”. While the addition of such quantitative information (e.g., 95% less harmful, as is used to describe e-cigarettes by Public Health England) [5] may help to make such claims more persuasive, such reductions can be difficult to quantify and express appropriately in short messages. Furthermore, challenges with numeracy literacy in the public may also interfere with the interpretation of such messages [30]. More research is needed on quantitative MRTP claims, including whether they would be more likely to increase product use among non-smokers.

This study also found some mixed responses to MRTP claim versions that emphasized the harms of smoking (i.e., “smoking is deadly”) or high numbers of chemicals in smoking. Consistent with a previous study [12], we found that such messages appeared to increase some smokers’ interest in quitting altogether, but not their interest in switching to snus or e-cigarettes. Some thought these types of messages would be “hypocritical” if coming from an industry source. More research is needed about the potential effects of this type of messaging strategy, particularly from health-related organizations/sources.

Regardless of the message type, our findings, consistent with others [12,18], suggest that the message source is important. Participants expressed skepticism about messages coming from industry (as well as “scientific research” perceived as industry-sponsored) and noted that some of the MRTP messages would be more meaningful if delivered from non-industry sources. As such, MRTP messages may be more effective if they are echoed by more trusted sources such as health professionals and organizations [31,32,33]. At the time of writing, e-cigarette information pages on the CDC’s website included some explicit statements that e-cigarettes are less harmful than cigarettes (though not safe) [17], though no similar statements were found for smokeless tobacco products. More research is needed to understand the potential effects of MRTP messages from non-industry sources.

Finally, it was encouraging to find that although young adult (YA) non-smoker participants understood the MRTP messages, they did not perceive that such messages would increase their product interest. This was due in part to their low interest in these products to begin with and skepticism of the claims. YA comments also suggested that some of the smoker-directed wording found in the claims (e.g., “if you smoke, consider this…”) made these claims feel less relevant and interesting to them. Although such wording may increase their length, future MRTP messages should consider including this type of smoker directed language to minimize appeal to non-smokers and unintended consequences, particularly if messages are placed in channels where non-smokers may be exposed to them (e.g., product packaging, point of sale, print ads).

Study limitations include the use of a small convenience sample of participants from a single geographic area at one point in time. Given the evolving nature of science and regulations of these products and their marketing, continued research on this topic area is warranted.

## 5. Conclusions

Quitting all nicotine products is the safest course of action. However, MRTP messages about snus and e-cigarettes may help promote harm reduction for some smokers who are unable or unwilling to quit nicotine. Although such messages may be understood, message acceptance may vary based on existing product beliefs (including competing/conflicting product information), MRTP message specificity, and perceived message source. As proposals for and use of MRTP messages may continue to move forward (by industry or other sources), additional research on MRTP message wording, framing, delivery, and effects may help to inform regulatory or organizational decisions about these messages, optimize their potential benefits, and mitigate unintended consequences.

## Figures and Tables

**Table 1:** Tested modified risk and exposure claims and wording variations, by focus group type (snus or e-cigarettes).

Message Type	Snus Groups	E-Cigarette Groups
Modified risk (MR) base claim	“If you smoke, consider this—switching completely from cigarettes to snus can greatly reduce your risk of lung cancer, mouth cancer, lung disease and heart disease.”	“If you smoke consider this—switching completely from cigarettes to vaping products can greatly reduce harms to your health.”
Variations to MR claim *		
With “completely replacing” instead of “switching completely”	“…Completely replacing your cigarettes with snus can greatly reduce your risk of lung cancer, mouth cancer, lung disease and heart disease.”	“…Completely replacing your cigarettes with vaping products can greatly reduce harms to your health.”
With “quitting smoking” instead of “switching completely”	“…Quitting smoking and switching to snus can greatly reduce your risk of lung cancer, mouth cancer, lung disease and heart disease.”	“…Quitting smoking and switching to vaping products can greatly reduce harms to your health.”
Without “greatly” wording	“…Switching completely from cigarettes to snus can reduce your risk of lung cancer, mouth cancer, lung disease and heart disease.”	“… Switching completely from cigarettes to vaping products can reduce harms to your health.”
With “much lower risk” instead of “greatly”	“…puts you at a much lower risk for lung cancer, mouth cancer, lung disease, and heart disease.”	
With “scientific studies” attribution	“…Scientific studies have shown that switching completely from cigarettes to snus can greatly reduce your risk of lung cancer, mouth cancer…”	“…Scientific studies have shown that switching completely from cigarettes to vaping products can greatly reduce harms to your health.”
With second statement emphasizing smoking harms	“… Switching completely to Snus can greatly reduce your risk of lung cancer, mouth cancer, lung disease, and heart disease.	“… Switching completely from cigarettes to vaping products can greatly reduce harms to your health.
	Smoking is deadly. Think seriously about switching.”	Smoking is deadly. Think seriously about switching.”
With explanatory second statement related to combustion and smoke	“…Switching completely to snus can greatly reduce your risk of lung cancer, mouth cancer…”	“…Switching completely from cigarettes to vaping products can greatly reduce harms to your health.
	Why? Because there is no burning of tobacco or smoke with snus.” (Version in groups 1,2,4)	
	OR	Why? Because there’s no tobacco burning or smoke with vaping products.”
	Why? Because there’s no smoke to inhale with snus.” (version in groups 3, 5, 6)	
Modified exposure (ME) base claim	“If you smoke, consider this—scientific studies have shown that snus products contain fewer harmful chemicals than cigarette smoke.”	“If you smoke, consider this—scientific studies have shown that vaping products release fewer harmful chemicals than cigarette smoke.” (groups 7, 8, 9)
		“…that vaping products expose users to fewer harmful chemicals than cigarette smoke.” (groups 10, 11, 12)
Variations to ME Claim *		
With “far fewer” or “a lot less” wording	“…Scientific studies have shown that snus products contain [far fewer/a lot less] harmful chemicals than cigarette smoke.”	“…Scientific studies have shown that vaping products expose users to [far fewer/a lot less] harmful chemicals than cigarette smoke.”
With high number of chemicals in cigarettes	“…Scientific studies have shown that snus products contain fewer harmful chemicals than the 7000 in cigarette smoke.”	“…Scientific studies have shown that vaping products [release/expose users to] far fewer harmful chemicals than the 7000 in cigarette smoke.”

* When presented to participants, all claim variations included the preamble “If you smoke, consider this”.

**Table 2:** Believability-related reactions to a modified risk claim for snus and e-cigarettes.

*A. Perceptions that claim is not specific enough; desire for more facts, statistics*
*Snus Groups*
“I feel like you don’t know for sure if this is factual and where the facts lie and what percent. So it’s not really that specific.” (female YA) “I’m very skeptical about any claims, because even the first statement, you know, ”can greatly reduce?” How do they—where are they getting that from?” (female smoker)
“…the sentence is clear to understand but it doesn’t tell you…how it’s safer…how snus does that, reduce the risk. If you were just reading that one sentence, you wouldn’t know how it does that.” (female smoker)
*E-cigarette Groups*
“…although I think it’s a pretty good statement, it doesn’t have any statistics about like how it can reduce” (male smoker) “So like they don’t tell you what harms they reduce…” (male YA) “I think that they should give like a small reasoning why, maybe” (female YA)
*B. Believability concerns based on existing product risk beliefs (that harmful/still harmful)*
*Snus Groups*
“You’re chewing on it. So it depends on what’s in it. If there’s tar and nicotine in that snus, now it’s more heavily in your mouth. I would think that it would increase the mouth cancer.” (female smoker) “It can still give you things in your mouth, I know that. So, that statement is not right.” (male smoker)
*E-cigarette Groups*
“Actually, in my opinion vaping and cigarettes to me is about that same….” (male smoker) “…nothing is gonna reduce harms to your health besides quitting, abstinence completely. So by saying it can greatly reduce harms to your health, it doesn’t make sense to me. “ (female smoker) “Because anything that contains nicotine is not safe. That’s how I know it’s not true. I don’t see how an electronic cigarette versus a non-electronic cigarette would be any safer if they both contain nicotine.” (female smoker)
*C. Believability concerns based on acute effects of vaping and EVALI outbreak (E-cigarette Groups)*
“…there are people dying due to vaping devices. So, with cigarettes, it is like it slowly and gradually kills you. But vaping devices…It’s just killing you. So the statement does not make any sense….” (female smoker) “The people that have died, basically, from JUULing. I know last week or this week there were five cases of hospitalizations and I feel like that’s just a lot quicker than people dying of cigarettes.” (female YA) “But knowing what’s going on in the media, I’m not a fan of—or will I ever be a fan of it.” (female smoker)

**Table 3:** Example quotes of reactions to specific wording and variations to a snus and e-cigarette modified risk claim.

*A. Positive perceptions of “greatly” qualifier*
“ I like greatly. Because it’s telling me it’s a more significant number. If I was to do what this says, that it would be a significant amount of instead of a little amount.” (female smoker, snus group) “I feel like it makes you feel like it has a big effect.” (male smoker, snus group)
*B. Negative/skeptical perceptions of “greatly” qualifier*
“I think, just the ‘greatly reduce’ just seems like it’s claiming a lot, that something is really gonna, like—boom, happen. The ‘lower risk’ is like, I feel like that’s a little more genuine…” (male smoker, snus group) “Feels more true.” [without greatly included]. (male smoker, e-cigarette group)
*C. Positive perceptions of attribution to “scientific studies”*
“I’d go out and buy that if it’s—you know, it says scientific studies. Still doesn’t give me the “how” that I want…. but it’s got that scientific studies.” (female smoker, e-cigarette group) “…that’d probably be the best way to say it, because it shows that there’s been studies behind it…” (male smoker, snus group)
*D. Negative/skeptical perceptions of attribution to “scientific studies”*
“Whenever I see anything that says the word scientific studies, I want footnotes or at least tell me—not necessarily like who did the study, but where’s it from, you know, ‘cause if you say that it’s from like John Hopkins or Yale or Harvard, I might take it a little more seriously ….” (male smoker, e-cigarette group) “I think you can find scientists to say whatever you want them to say, to be honest, nowadays.” (male smoker, snus group)
*E. Positive perceptions of “Smoking is Deadly” addition to MR claim*
“It’s very honest and it’s real.” (female smoker, snus group) “Pretty scary message. It will make me scared to thinking about maybe not switching but just quitting completely.” (female smoker, snus group)
*F. Negative/skeptical perceptions of “Smoking is Deadly” addition to MR claim*
“… you say smoking is deadly, but you still want me to switch to something else instead of quitting. So that would just completely lose their credibility.” (female smoker, e-cigarette group) “I find that incredibly disingenuous coming from a tobacco company, especially with one of its products that contains carcinogens. It still runs those risks.” (male YA, snus group)

**Table 4:** Participant reactions to a message about modified exposure to chemicals from snus or vaping products compared to cigarettes.

*A. Clear communication that Snus/Vaping is less harmful*
“There are less chemicals used in vaping. If you vape, it would be less harmful than smoking.” (female smoker, e-cigarette group) “Definitely the wording. They used the word fewer harmful chemicals, so I would just assume that it was less harmful than smoking.” (female YA, snus group)
*B. Perceptions that claim is vague about chemical type/quantity and harm reduction*
“If cigarettes have 10 harmful chemicals, Snus maybe might have 5, but they might have more of those 5 chemicals than the 10 in the cigarette smoke.” (female smoker, snus group) “Like, maybe it only has one harmful chemical, but it has, you know, like, a lethal dose of the harmful chemical, okay?” (female smoker, snus group) “…just because it’s less doesn’t mean it’s better. Like you can have a million bad things, having two less bad things is a little better but it’s not that big a difference.” (male YA, snus group) “…like they’re saying fewer harmful chemicals, but they’re not saying which chemicals…. It could be different chemicals that are all classified as harmful, and worse still…” (female YA, e-cigarette group)
*C. Skepticism about e-cigarette claim believability because of the chemical nature of e-cigarettes*
“Because it’s factually inaccurate. Because cigarettes have tobacco, tobacco is a plant, that’s natural. Vaping and e-cigarettes are all chemicals, so how can it have fewer chemicals…” (male smoker, e-cigarette group) “… if you roll your own cigarettes, you’re just putting tobacco into paper and you’re lighting it. If you’re vaping, who knows what chemicals are in them? So I don’t know even that it makes that much sense.” (female smoker, e-cigarette group)
*D. Perceptions that reference to 7000 chemicals in cigarette could be effective:*
“That’s really scary. That’s gonna really make somebody think. ‘Cause I guarantee you the average person who smokes is unaware that there’s 7000 chemicals.” (female smoker, snus group) “Once they see the number, I think people would stop… Once they see the number, they’re like, my God, less than 7000. Amazing.” (female smoker, e-cigarette group)
*E. Perceptions that reference to 7000 chemicals in cigarette could be less effective:*
“…far fewer, even if it’s half, it’s still a lot of chemicals.” (male YA, snus group) “…like what is ‘far fewer’? That could be 4000 harmful chemicals, 3000, 5000. So I think it makes me question the threat of snus even more.” (male YA, snus group) “I honestly thought this one was like worse because, in the past one I didn’t think about harmful chemicals and then like this one makes me think about harmful chemicals, so it’s like why would I pick this up if it has—if it still has harmful chemicals?” (male YA, e-cigarette group)

## Supplementary Materials

The following are available online at https://www.mdpi.com/1660-4601/17/18/6807/s1, Table S1: Claims proposed in MRPT applications to the US Food and Drug Administration for smokeless and heated tobacco products.
