# Supplementary material for: Smokers’ and Young Adult Non-Smokers’ Perceptions and Perceived Impact of Snus and E-Cigarette Modified Risk Messages
Source: Int J Environ Res Public Health. 2020 Sep 18;17(18):6807. doi: 10.3390/ijerph17186807 (PMC7558440; doi:10.3390/ijerph17186807)
Supplement: Supplementary file 1 [file ijerph-17-06807-s001.pdf]

Supplementary Table S1. Claims proposed in MRPT applications to the US Food and Drug Administration for smokeless and heated tobacco products.

Claims Proposed in Application for General Snus:

1. "Using General Snus instead of cigarettes puts you at a lower risk of mouth cancer, heart disease, lung cancer, stroke, emphysema, and chronic bronchitis." (main proposed claim)
2. "Using General Snus instead of cigarettes would significantly reduce harm and the risk of certain tobacco-related diseases to individual tobacco users."
3. "No tobacco is totally safe, but using General Snus instead of cigarettes puts you at a lower risk of chronic lung disease and other tobacco-related ailments."

Claim Proposed in Application for Copenhagen:

1. "IF YOU SMOKE, CONSIDER THIS: Switching completely to this product from cigarettes reduces risk of lung cancer."

Claims Proposed in Application for Camel Snus:

1. Smokers who switch completely from cigarettes to Camel Snus can significantly reduce their risk of lung cancer, oral cancer, respiratory disease, and heart disease. ("KEY CLAIM")
2. Smokers who SWITCH COMPLETELY from cigarettes to Camel Snus can greatly reduce their risk of lung cancer, oral cancer, respiratory disease, and heart disease. ("KEY CLAIM")
3. Smokers who SWITCH COMPLETELY from cigarettes to Camel Snus can greatly reduce their risk of lung cancer and respiratory disease. ("KEY CLAIM")
4. Smokers who use Camel SNUS instead of cigarettes can significantly reduce their health risks from smoking.
5. Scientific studies have shown that Camel SNUS contains fewer carcinogens than cigarette smoke.
6. Scientific studies have shown that Camel SNUS contains less of the harmful chemicals than cigarette smoke.
7. No smoke means...
    - No hassle
    - No lingering smoke smell
    - More freedom
    - Fewer carcinogens
    - Less risk for you and those around you
8. Switching to SNUS means ...
    - Less of the harmful chemicals found in cigarette smoke
    - Less risk for you and those around you
    - No lingering smoke smell
    - Hassle-free tobacco
9. Swap the smoke for more freedom and less risk.
10. No smoke. Less risk. Choose SNUS.
11. NO SMOKE = LESS RISK

Claims Proposed in Application for IQOS:

1. "Scientific studies have shown that switching completely from cigarettes to the iQOS system can reduce the risks of tobacco-related diseases".
2. "Switching completely to iQOS presents less risk of harm than continuing to smoke cigarettes".
3. "Scientific studies have shown that switching completely from cigarettes to the iQOS system significantly reduces your body's exposure to harmful or potentially harmful chemicals".

With additional "clarifying information":

- "The IQOS system heats tobacco but does not burn it."
- "This significantly reduces the production of harmful and potentially harmful chemicals."
